# Start of the Season in a Seasonal Work Context: A Better Understanding of the Difficulties Experienced by Seasonal Workers in the Food Processing Industry for the Prevention of Work-Related Musculoskeletal Disorders

**DOI:** 10.3390/ijerph21080997

**Published:** 2024-07-30

**Authors:** Audrey Goupil, Marie-Eve Major

**Affiliations:** 1 Faculty of Physical Activity Sciences, University of Sherbrooke, Sherbrooke, QC J1K 2R1, Canada; 2 Department of Industrial Relations, University of Laval, Quebec City, QC G1V 0A6, Canada; 3 CINBIOSE Research Centre, Montreal, QC H3C 3P8, Canada

**Keywords:** work-related musculoskeletal disorders, seasonal work, work activity analysis, occupational health, physical risk factors, organizational factors, psychosocial factors, ergonomics, return to work, meat processing

## Abstract

The specific period of the start of a new working season and a return to work after the off-season seems to be a critical moment for the musculoskeletal health of seasonal workers. This study aims to identify the difficulties and working conditions encountered by seasonal workers in this particular period of the working season which may increase the risk of work-related musculoskeletal disorders (WMSDs). An in-depth ergonomic work activity study, combined with a multiple case study of eight seasonal workers from a meat processing facility, was conducted. Various interviews (*n* = 24) and observations of work activity, organization, and production (*n* = 96 h) were held at different moments (off-season, return to work at the start of the season, and during the season). Critical work situations exposing workers to WMSD risks emerged and highlighted a diversity of difficulties, such as accomplishing work activity involving strong physical strain and a significant and underestimated mental load, and having to rapidly develop new skills or re-learn working strategies after a long off-period. The study findings have implications for developing actions to prevent WMSDs that target working conditions and support a return to work for returning seasonal workers and a start of work for new seasonal workers, and to address work disability in this context.

## 1. Introduction

Work-related musculoskeletal disorders (WMSDs) are considered a major public health challenge in many industrialized countries [1]. Seasonal workers are particularly affected by this problem [2,3,4]. Seasonal work is an integral part of employment markets in many countries, and these workers are involved in various industry sectors (agriculture, forestry, food processing, etc.). In Canada, the proportion of seasonal work sits between 2% and 3% of overall employment [5]. As many as 33% of seasonal workers come from the goods production sector, and of these, 20% are employed in the sub-sector of food processing [6].

The scientific literature interested in seasonal work paints an unsettling picture of working conditions that create situations involving a great risk of WMSDs for these workers. Some of the challenging conditions reported by seasonal workers include long working hours, many consecutive working days without a day off, a high intensity of work, and repetitive tasks, as well as short working cycles [4,7,8,9]. Furthermore, the work is often conducted in conditions involving extreme temperatures (cold or hot) and elevated noise levels, the frequent and prolonged use of vibrating tools, and a working rhythm dictated by the speed of the machinery involved [2,8,9,10,11]. The physical strain and the organizational, environmental, and time-related constraints are generally “endured” by seasonal workers “because they only last for the length of the season” [4] (p. 95).

Seasonal work is characterized by the alternating pattern of a “working season” and an “off-season” [2,4,8]. During the working season, tasks are often conducted in unpredictable, fluctuating, and intense conditions [2,9,11,12] that impact the musculoskeletal health of the workers [4,13,14]. According to a literature review of this issue [13], seasonal workers report WMSDs in several body regions (ex: back, shoulders, knees, neck, etc.), and these reports come from various industry sectors (ex: agriculture, food processing, fishing, forestry, etc.). WMSDs have been reported as a major health concern among seasonal workers. In particular, the work of Lipscomb et al. [15] in the fishing industry and that of Schweder [16] in the agricultural processing industry have revealed a higher prevalence of musculoskeletal symptoms and higher rates of work-related injuries and serious injuries among seasonal workers performing the same or similar jobs as their permanent counterparts. Additionally, the WMSDs reported by seasonal workers tend to be chronic, despite the existence of an off-season [4,14,17].

At present, very few studies have been carried out to better understand how the start of the working season unfolds. This period involving the start of a working season and/or a return to work after an off-period deserves a closer look to better understand WMSDs and prevent disability in seasonal workers, and also with consideration for both new seasonal workers and returning or repeat seasonal workers who could experience this particular period differently. The question is even more pertinent when considering that seasonal workers tend to consider that musculoskeletal pain is an integral part of their work [4,18], an assumption that is also known to be shared by the employers of seasonal workers [10].

The aim of this study was to identify difficulties and working conditions encountered by seasonal workers in a meat processing facility, in particular at the start of the season and which may increase WMSD risk.

## 2. Materials and Methods

### 2.1. Study Design

The methodology of this study is based on an ergonomic approach to work activity analysis [19,20] and relies upon a descriptive multiple-case study design [21]. A case was defined as a seasonal worker’s working in a food processing plant. We selected generally similar cases, but with certain specific distinctions (ex: male or female, years at the plant, different workstations) in order to obtain a wealth of in-depth data and in accordance with case study recommendations [21,22]. In this regard, the following criteria were used to select the participants: (a) they must work in the same plant in the food processing sector, (b) work in the production sector, (c) work on a seasonal basis, and (d) speak French. Participants were recruited via non-probability purposive sampling [23]. In total, eight seasonal workers (four women and four men) coming from a meat processing facility and occupying a variety of production workstations were selected. This sector of activity is characterized by a lower level of education among workers [24,25], which is consistent with the characteristics of the participants, who had no educational or professional qualifications. A case study makes it possible to enrich theories (analytical generalization) rather than to obtain frequencies and statistics (statistical generalization) [21].

Table 1 presents a description of the cases. Four men and four women participated in the study. The mean age of the participants was 18.6 years (minimum = 15 years, maximum = 28 years), as a large proportion of Canadian seasonal workers are aged between 15 and 24 years (44.3% of seasonal workers in Canada, 2022) [5]. In Quebec, the minimum working age is 14 years old [26], and no employer may have work performed by a child that is disproportionate to the child’s capacity, or that is likely to be detrimental to the child’s education, health, or physical or moral development [27]. The workers had between zero (first-time seasonal worker) and four seasons (returning seasonal workers) of prior experience in the facility. These seasonal workers were employed in production during the working season (May–June to August) and were at school during the off-season. The participants worked 37.5 h per week, and none of them worked overtime.

All the workers who participated in the study reported musculoskeletal pain in the neck, lower back, shoulders, arms, wrists, and feet during the first four weeks of their working season, and none of the participants reported having any pain during the two weeks prior to the start of the season. Participation was voluntary, and all participants were informed about the study and gave their consent to participate.

### 2.2. Methods and Data Collection

**Figure 1:**
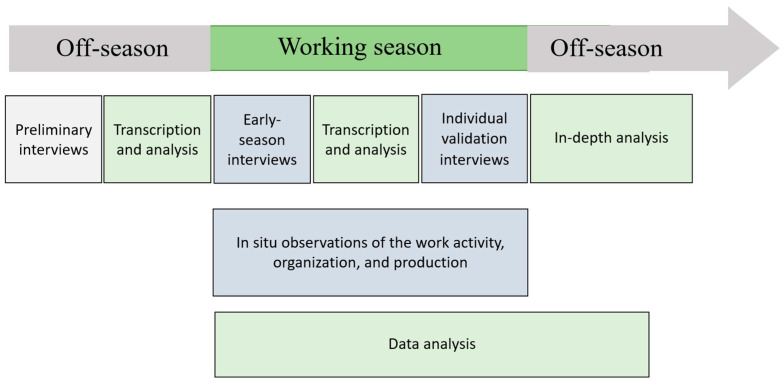
Data collection and analysis process.

Data were collected using a variety of methods to promote an in-depth understanding of real work, in order to identify difficulties and working conditions encountered by seasonal workers that may increase WMSD risk. Figure 1 shows the data collection and analysis process. The use of multiple sources of evidence (various interviews, observations of work activity, organization, and production) was carried out in a complementary way between each of the data collection methods, in order to compare the data obtained by each method, as well as to obtain and develop new data. The iterative process between data collection and analysis contributed to the interaction between various methods and enabled the validation of results, while ensuring depth and richness. This process is one of the characteristics and strengths of the ergonomic approach centered on work activity [19,20] and was also used in accordance with recommendations for increasing the validity of case study results [21].

A total of 24 in-depth, semi-structured interviews were conducted with the participating workers at different moments [28]. The preliminary interviews took place before the working season began. The interview guide was based on the one of Major et al. [29] specific to the context of seasonal work. The questions addressed during the interview concerned (a) the person and his/her characteristics (e.g., age, experience, training, etc.), (b) his/her state of health, and (c) his/her occupations during the off-season (e.g., physical activities, leisure, jobs, studies, etc.). In the first two weeks of work, early-season interviews were held. The interview guide used [20] was based on the work activity-centered model, which focuses on understanding work activity as performed, work organization, work variabilities, difficulties encountered, determinants, and possible consequences of work on both health and production. These interviews also involved the use of a body map. Participants were asked to identify the body regions and intensity of their pain, as well as the strategies developed to manage pain, by filling in a body map [14,30,31]. The body map used comprised 33 body regions, in order to obtain a precise localization of the body regions solicited by the work, and corresponded to that used in previous ergonomic research [29,31,32,33,34]. In addition, a numerical scale was used to identify the degree of pain (1 to 5; “No discomfort” to “Unbearable discomfort”), and short descriptions were associated with each degree of pain to ensure consistency in the interpretation of pain levels [30]. The body map was used during the interviews to facilitate verbalization of the difficulties encountered at work. The early-season interviews also allowed the targeting of moments and work situations for the observations. All interviews were recorded, transcribed verbatim, and lasted between 45 to 70 min.

In situ observations of each participating worker were also used to collect more specific data on work activity, organization, and production [19,20] and were conducted at different moments at the start and during the season. A total of 96 h of observation were carried out in the workplace during the six weeks of the season. More specifically, of this total number of observation hours, each participant was observed between two and four times over consecutive periods of three to five hours each time. The observation periods included taking notes (paper and pencil), photos, and video recordings. Between four and six hours of filmed observation (depending on the worker’s schedule and workstation occupied) were conducted for each participant and recorded on video, for a total of 39 h of video recordings. Handwritten notes, photos, and videos were used to document tasks, different operations, strategies used to accomplish tasks, working rhythms (ex: the number of chickens hung, the length of the work cycle), movements, postures, tools used, communication and interaction with colleagues, work variability, and any other details deemed pertinent (ex: number of workers on the production line, change of product on the line, machinery breakdown, etc.) of the work organization [19,20]. Additionally, when possible, real-time spontaneous verbalizations were solicited to better understand the work activity.

Finally, following a preliminary data analysis, individual validation interviews were held with each participant at the end of the working season. The data analyzed came from the pre-season and early-season interviews and observations of the work activity, organization, and production. The goal of these interviews was to validate the preliminary results and deepen the data interpretation if needed. Thus, for each participant, video sequences were selected on the basis of the representativeness of the work carried out, the difficulties encountered, and the potential determinants. Based on these data, an interview guide was drawn up for each participant, to clarify any necessary information to better understand the work activity, difficulties encountered, and determinants and to verify interpretations. During the individual validation interviews, the video sequences selected for each of the workers were shown to them, and the participants were asked to describe ‘how’ they carried out their work and the ‘why,’ as well as to describe the difficulties encountered and their causes [20]. Based on the video sequences, the participants were also asked about the sources of variability in their work, particularly during the first weeks of the season. Validation interviews were also recorded, transcribed verbatim, and lasted between 50 to 70 min.

### 2.3. Data Analysis

All recorded interviews were fully transcribed. A qualitative content analysis of the interviews was conducted [35] and was also based on the work activity-centered approach [20]. The body of data was read multiple times to acquire an overall understanding of the contents and give a sense of immersion. The interview content was then hand-coded to identify any element that indicated the difficulties experienced by workers and their determinants. The difficulties and the determinants (the codes [micro level]) were then regrouped into critical work situations involving a risk of WMSDs that exist at the start of the season (themes [macro level]) [36].

Video and photo data and handwritten notes taken during the observation periods were analyzed to help develop a detailed understanding of the work activity, to further enrich the understanding of the seasonal workers’ difficulties and to identify their determinants.

Both intra-and inter-case analyses were conducted [21]. Inter-case analysis [21] made it possible to account for the uniqueness of each case and to gain an understanding of the real work involved [20]. Inter-case analysis [21] aims to compare the cases with each other and to gain a better understanding of the sources of variability in the work, with a view to identifying the difficulties and working conditions encountered by seasonal workers. These analyses led to the identification of three critical situations at risk of WMSDs (difficulties and working conditions).

Several meetings were held, as well as much back and forth between the researchers, for the data extraction and coding, in order to ensure consistency with the aims of the project and to produce a consensus on the codes and themes and their meaning. The findings presented in this article stem from the qualitative content analysis of the triangulation of these in-depth interview and observation methods. The guidelines Standards for Reporting Qualitative Research [37] and case studies [21] were used to report the information pertaining to the study.

## 3. Results

### 3.1. Working Conditions

The work in this poultry-processing facility takes place on a semi-automated production line. The workers are situated at different points along conveyor belts passing along a significant quantity of raw materials, roughly 4800 chickens per hour. The working rhythm is dictated by the speed of the conveyors and the speed of colleagues situated upstream on the production line. Interactions between workers are limited.

Participants worked 7.5 h a day, Monday through Friday, and none of the participants did any overtime. Every 20 min, workers on the same production line rotate workstations. According to the observations, all the workstations share similar postural constraints and an intense working rhythm. Additionally, work in this plant takes place in cold and damp conditions, with drafts in the areas near the refrigerated rooms.

### 3.2. Difficulties Encountered by Seasonal Workers at the Start of the Season and Determinants

The study results highlight differences in terms of how the early season unfolds, depending on how many years of experience a worker has. When workers with prior experience return to the production line after the off-season, they are assigned to various workstations. Their assignments are given immediately, and there is no adjustment process or progressive return to work. When new workers arrive at the start of the season for their first experience at the processing facility, they are paired up with a permanent worker who is meant to integrate them onto the production line. There are no formal modalities or rules around this integration process, and each permanent worker in charge of integrating a new seasonal worker proceeds as he or she pleases. Three critical working situations involving WMSD risks illustrate the work-related difficulties these seasonal workers encountered at the start of their season and their determinants. The following will further describe the three main critical working situations involving a risk of WMSDs that the study identified in light of the difficulties encountered by both the returning seasonal workers and the new seasonal workers and the determinants of those challenges.

#### 3.2.1. Significant Physical Strain as Soon as the Season Begins

The results show that in the first days of the new season following a roughly nine-month off-season period, seasonal workers (returning and new) execute short-cycle tasks in which a series of operations are accomplished in roughly 2 to 10 s. For example, at the hanging a chicken workstation, 25 to 27 cold chickens are handled per minute. The rapid working rhythm is set by the speed of the conveyors, and the seasonal workers must get used to it immediately when the season starts; workers reported that it was difficult to match their speed to meet the requirements. About this, the workers say

“The hooks go fast, you can’t miss your movement, you have to be just as fast, (…) keep up with the rhythm. This is a problem because it’s often this speed that also causes pain. When it goes fast, you feel like it’s going to be really difficult (Verbatim extracts from examples of participant difficulties. The extracts are a free translation from the original French transcripts.).”

[T1, returning seasonal worker]

“[When there are many chicken breasts], you’re going to try to grab them, but anyway, they keep going, they keep going, you catch one, you catch one, but then you miss one. You’re not going to be able to get all of them.”

[T6, new seasonal worker]

**Figure 2:**
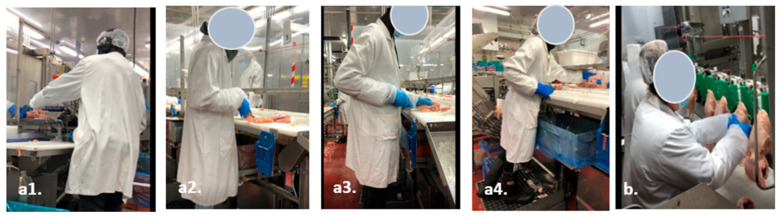
Examples of significant physical strain (**a1**–**a4**) Different steps for the task of trimming chicken breasts with a knife and (**b**) task of hanging a chicken).

The results also demonstrate that there is a lot of repetition under rapid conditions. The work essentially involves repeating the same gestures for particular articulations (wrists, shoulders, back, neck) over and over again and in a wide range of motion (80–90° flexion of the left shoulder, approximately 10–20° extension of the right shoulder, ulnar deviation of the right wrist, approximately 20° flexion of the body trunk, 30° flexion of the neck, static upright stance) as illustrated by Figure 2.

All participants reported that having to execute repetitive movements in awkward postures caused significant musculoskeletal pain, especially just as the season started; this pain affected their productivity, as the following testimony from one of the workers expresses:

“On the second [day of the season] I was on the [production] line all day, (…) the repeated movements hurt. So my wrists and my arms, they hurt, (…) making it a little harder to keep up with the work.”

[T8, new seasonal worker]

Starting out the season after nine months of off-season with work involving significant musculoskeletal strain is a big issue that the seasonal workers reported.

#### 3.2.2. Quickly Learning/Relearning Practical and Preventive Skills

As soon as the season begins, workers take their position on the production line and must accomplish movements that are hard for the body, in contrast to the off-season, when these seasonal workers are in school. Workers indicated that the need to immediately learn or relearn know-how and the expectation to be instantly efficient was difficult, especially because of the wide gap between their fluency with the movements and the working expectations immediately at the start of the season. Being unfamiliar with the quality and quantity requirements, unaccustomed to new working requirements compared to the season before, or not yet used to how to work to meet the requirements meant the workers were constantly worried about the work they were doing and adjusting their working speed, all of which created a high mental load. About this, one of the workers reported

“When I went back to work at the start of the season, it had been a long time since I’d worked, and it felt like the line was moving even faster. It doesn’t make sense how fast it goes. There’s no time at all to take a break. (…) [You] have to go faster and faster. It’s like we are always worried, “is that how you do it, am I missing something?” ”

[T2, returning seasonal worker]

It is clear that for these returning seasonal workers, the off-season leads to the loss of certain “habits”, “familiarity”, and “skills” vis-à-vis the required tasks. Also, workers report that during the same period, the manufacturing processes and work organization may change, but they are not or rarely informed about the changes when they start working at the facility again. After the off-season, they report finding it difficult to meet the expectation of immediate professional efficiency. The requirements do not offer any leeway that would make it possible to develop the know-how required or relearn certain skills, as well as strategies for managing the pain. A returning seasonal worker reported

“It’s very much an abrupt return to work. It’s like I’d lost the habit of doing the movements I’d done every day, which meant I had to go from never doing that to doing it all day long, which means that… in the beginning [of the season], it was definitely rough … for my injuries. At the beginning [of the season], it was like I was a bit lost. It just went a little too fast.”

[T2, returning seasonal worker]

These results highlight the difficulties that repeat seasonal workers experience when returning to work after the off-season, such as having to rapidly get used to the work and its requirements, as well as relearning skills and working strategies.

In terms of new seasonal workers, they are either unfamiliar or not very familiar with the work that must be done, nor do they know how to do the work at the start of the season. The results made it clear that the way in which know-how was transferred, as well as what know-how was transferred, to new seasonal workers was highly varied. The latter reported integration times varying between 15 min and five consecutive days. Additionally, as the following excerpt shows, they were given very little information about the work that must be done at each workstation:

“They tell you only the essential. You have to remove all the bones. They won’t show you how to remove the bone. You have to figure it out and cut.”

[T7, new seasonal worker]

“They didn’t really show us how to hang the chicken. There is a specific way to do it so you don’t hurt your arms. And I didn’t know this, as I ended up hurting myself, it hurt at the end of the day. And it went so fast…. For the first time on the line, you feel a bit lost because it’s going too fast. (…) You don’t understand why you’re doing it so badly, while the others beside you find it super easy.”

[T8, new seasonal worker]

There is clearly a desire on the part of the new seasonal workers to receive information on “how” to accomplish the tasks, especially in terms of know-how and working strategies. New seasonal workers report this lack of information as leading to musculoskeletal pain and difficulties during the start of the season, like having to quickly develop new skills and execute unfamiliar movements.

#### 3.2.3. Rapid Adjustment to Working Complexity: A Non-Negligible Cognitive Activity

Although at first the different production line tasks seem simple, our results show the work is more complex. The complexity arises because the raw material comes through in unpredictable ways, and there is a need to anticipate what will come on the conveyor and how the characteristics of the raw material and the products vary, as well as having to concurrently execute different tasks and operations. Workers report a specific need to be aware of carcasses, undesirable chicken parts, chicken position, etc., to avoid slowing down production, as well as anticipate a mechanical problem. To keep the work flowing, workers must quickly adjust to unexpected situations, by adding new operations—for example, like grabbing a carcass from the conveyor to keep it from overflowing. The need to adjust so quickly to react to unexpected situations or anticipate any problems was documented as being difficult for seasonal workers, especially at the beginning of the season.

The results also indicate that these different variations, which begin right away at the start of the season, require a lot of focus and concentration, as the following statement from a returning seasonal worker illustrates:

“When you come back [at work], how your memory gets working, I did that, and then at the beginning, I felt that it was more difficult to learn again…. When I start working again at the beginning, I have the sense that it feels somewhat different. It changes depending on the products. With the rounds [chicken pieces], there was a lot more to do. For the rounds [chicken pieces], you had to make them flat [lying flat in the bag]. And then get rid of some of the air that was inside the bag so it wouldn’t cause a problem. And then to see if the box had no defects. The movement to place the rounds, that [also] varied.”

[T1, returning seasonal worker]

A new seasonal worker related that having to concurrently perform multiple tasks and operations made the work more complex:

“Mostly, you have the pedal, that means that you’re hanging like everyone else, except you also have to watch the pedal down below, which is what opens the door to let the chickens slide down and come onto the belt [the conveyor]. (…) You can’t ever not have chickens in front of you [the conveyor]. Sometimes you hit the pedal while forgetting about the other thing or you do the other thing [hang the chicken on the hook] while forgetting the pedal. Sometimes, there aren’t any chickens and the others are like, “come on, send the chickens”

[T6, new seasonal worker]

This comment shows that paying attention is essential to completing the task. Workers must also evaluate and anticipate the quantity of chickens to be sent onto the belt and the quantity required for the other workers, and they must do this before there are no more chickens on the conveyor. To do this, the workers observe and analyze the quantity of chickens on the conveyor, in order to decide whether to have more sent down or not. The decision affects the other workers as well.

These results illustrate the difficulties in terms of evaluating and rapidly identifying certain characteristics about the raw material in order to make proper decisions, anticipate quantities for production and colleague’s needs, and rapidly adjust for unexpected situations and foresee other uncertainties.

## 4. Discussion

The results highlighted three main critical working situations which may increase WMSD risks that seasonal workers are confronted with when they return to work after an off-season of nine months: (1) dealing with working conditions that impose significant physical strain, (2) having to quickly develop new skills or re-learn strategies, and (3) being confronted with work that requires a significant but underestimated mental load. These results made it possible to describe a variety of difficulties linked to the work, as well as the determinants of the situations which may increase the WMSD risks that these workers must negotiate. In this way, this article contributes to a shared representation of the difficulties experienced by repeat seasonal workers upon their return to work after the off-season period and by new seasonal workers just beginning their season, which seems to be a key issue in improving the efficacy of WMSD prevention [38]. Until now, very few studies have focused in a specific way on the particular period of seasonal work that combines the start of the season and the return to work, and even less so in terms of understanding work activity as a way of preventing WMSDs. Via a work activity-centered approach [19,20], these results contribute to a better understanding of this understudied work context and the situations that carry WMSD risks that seasonal workers must negotiate as soon as the season begins.

Our analysis revealed several determinants coming from the work environment that contributed to the difficulties experienced by the workers. The determinants were especially related to the high working expectations in place at the start of the season after a long off-season period, working rhythms imposed by the speed of the machines involved, a significant volume of production required immediately as work started up again, an assigned rotation through similar workstations all involving awkward postures, and an intense working rhythm, as well as having to work in a difficult physical setting (cold, damp, drafty). These determinants agree with those raised in other studies looking at the context of seasonal work [2,8,39,40,41] and in particular in the sector of food processing [9,10,11,14,29].

Additionally, this study highlights the constraints linked to the process of returning to work after an off-season period and to the methods of integrating seasonal workers at the start of the season, which are not formalized and even quite limited. These situations are recognized as contributing to an increased risk of WMSDs [42]. More specifically, our study provides new knowledge on the difficulties experienced at the start of the season by returning seasonal workers, as well as new seasonal workers. Notably, for repeat seasonal workers, returning to work after the long off-season occurs in the exact same conditions as when they stopped working nine months earlier. Furthermore, these seasonal workers are not necessarily aware of the changes that may have been implemented in the meantime in, among other things, manufacturing processes and work organization. When returning to work after the off-season period, these workers find it difficult to quickly re-implement their skills and strategies or rapidly refamiliarize themselves with the work and its demands. Workers are expected to be immediately professionally efficient as soon as the season begins, but the start of the season conditions (limited integration procedure, high production expectations, speed requirements, etc.) end up narrowing the margin of maneuver [19,43] that seasonal workers have, making it difficult for them to find a balance between their health and their performance. Work organizations that are conducive to preserving health are those that generally increase a worker’s margin of maneuver in dealing with unforeseen, fluctuating situations, in order to give and support the opportunity to readjust their activity [20,43,44]. Furthermore, increasing the margin of maneuver is considered a factor for preventing the risk of WMSDs [45].

In this perspective, it would be important to look into the conditions involved in the return to work at the start of the season for returning seasonal workers as a way to prevent WMSDs and disability. The principles underlying the progressive return to work process that follows a WMSD-related absence [46,47] could be useful in rethinking the integration process of returning seasonal workers at the start of the working season. These principles stipulate that adapting the environmental constraints to the working capacity through a progressive exposure to work, while ensuring a balance between health and production requirements, should promote a return to regular work [46,47]. The conditions being offered when work starts up again after the off-season should offer enough margin of maneuver to help workers reappropriate skills (know-how) and strategies (ex: request a change of position in the case of any pain, adjust the working speed to a rhythm the worker finds suitable, modulate exposure to working conditions, ensure a form of collaboration or help with a permanent worker, etc.) [29,43,48]. These types of conditions would better equip workers to optimally adjust their skills to each working situation, to develop regulation strategies that promote a healthy and sustainable work environment [9].

The results of our study also suggest significant difficulties linked to the integration of new seasonal workers. As soon as they arrive, they are subject to rapid working rhythms under heightened production expectations in terms of volume and speed. These types of conditions impact learning [49], since at the start of the season, new seasonal workers are not familiar with the tasks needing to be done nor how to do them. Situations like this contribute to the difficulties identified in this study, meaning having to quickly develop new skills and execute unfamiliar work-related movements. These are difficult working conditions (fast working rhythm, large production volume, non-formalized integration processes and content) that can have significant repercussions on occupational health—in particular, an increased risk of accidents and disabilities [49], especially for temporary workers [50,51]. Following our observations and with a goal of preventing WMSDs and disability in new seasonal workers, the integration process should be re-evaluated, and favorable working conditions implemented from the moment the season starts, even if these workers are only going to be employed for a short period of the year. Something that can help integrate new workers would be to consider the complexity of tasks and the variability of the working situations [49], especially through the identification and explanation of visual and tactile cues of the work with workers [42]. Indeed, our results reveal that the work is highly complex, and new seasonal workers need to receive more information on “how” to execute their tasks, particularly in terms of operations and strategies. In this sense, integration should target the development of cues and involve a progressive exposure to the different working complexities, by controlling some of the working conditions during the learning period [49]. For example, this kind of progressive learning could be implemented in the form of a back and forth between the production line and controlled conditions outside of production, by modulating the working rhythms, the volume of production, the variability of characteristics of the raw material, or even the assigned rotation to different workstations, all while contributing to production. An integration of this nature would help develop the workers’ autonomy under real-work conditions and foster a greater diversity of skills, so that when later faced with complex and/or unexpected situations, they have a larger tool kit to draw from. This would help new seasonal workers build their own skills (adapted to both the context and their personal characteristics), with a goal of meeting work requirements, while ensuring a balance with their health.

In light of these various difficulties and the determinants faced by new as well as repeat seasonal workers, it is essential to examine the factors that promote and those that challenge the implementation of an intervention aimed at improving working conditions for the prevention of WMSDs in a seasonal context. To date, very few studies have focused on interventions aimed at preventing WMSDs in a seasonal work context, and the processes of interventions deployed in such a context are virtually unknown [13]. And yet, such a context is a difficult reality for workers, but also inescapable for companies working with natural resources whose availability and lifespan may be temporally limited and dependent on climatic and meteorological conditions. Such a context can pose challenges for the implementation of a WMSD prevention intervention. Moreover, the issues involved in WMSD prevention seem to be poorly understood or unknown to companies [2], and it is customary to believe that WMSDs can be attributed to a single cause [10]. The issue of mobilizing the various key players (workers, employers, unions, industry representatives, etc.) would need to be at the heart of such an ergonomic intervention approach. Future studies evaluating the implementation of WMSD prevention interventions in a seasonal context and focusing on the mobilization of the various key players, as well as on the process of co-constructing ways of improving working conditions, would be of relevance. Such a study is currently underway [52].

## 5. Limitations

This study was interested in the difficulties of eight seasonal workers in a meat processing facility. Seasonal workers are recognized as a heterogeneous group composed of distinct seasonal worker profiles and different activity sectors [4,53]. This study focused on the category of “persons engaged in seasonal work for supplementary purposes” [53], defined as workers who return to work from time to time in the same company and who do this work by choice, especially as a way to reconcile work and education. The results of this study are connected to the characteristics of these seasonal workers and to the working conditions of this particular facility. Despite its limitations, this qualitative study achieved a systemic work activity analysis, with in-depth interviews and observations that generated a rich understanding and detailed descriptions of the difficulties and concerns that are likely experienced by other seasonal workers who work under similar organizational conditions.

## 6. Conclusions

In conclusion, working conditions at the start of the season could be a big issue for seasonal workers returning to work after the off-period, as well as for new seasonal workers starting their first season. Several determinants coming from the work environment, such as rapid working rhythms under expectations of high production (both volume and speed) and an immediate expectation of developing or re-appropriating professional skills that are somewhat complex because of how greatly the work varies and how rapidly workers must adjust to unexpected situations or anticipate uncertainties, contribute to important difficulties experienced by seasonal workers as soon as the season begins. Prevention actions must consider this critical period and address the physical strain, as well as the organizational, environmental, and psychosocial constraints, of seasonal worker musculoskeletal health.

## Figures and Tables

**Table 1:** Description of the 8 cases.

	Sex ^1^	Age	First-Time Seasonal Worker	Returning Seasonal Worker	Years of Experience in the Plant (and in the Sector)	Start of Work Date	End of Work Date	Work Schedule	Workstations					
									Hanging	Trimming	Cutting Table	Packing	Palletizing	Freezing
T1	M	18		X	3	2 June 2021	6 August 2021	7:00 to 15:30	X	X	X	X	X	
T2	M	20		X	4	31 May 2021	20 August 2021	7:45 to 16:15				X	X	X
T3	F	20		X	1	23 June 2021	6 August 2021	7:45 to 16:15	X	X	X			
T4	M	16		X	1	28 June 2021	19 August 2021	7:00 to 15:30				X	X	
T5	F	16	X		0	28 June 2021	20 August 2021	16:00 to 00:00	X	X	X			
T6	M	15	X		0	25 June 2021	20 August 2021	7:00 to 15:30	X	X	X			
T7	F	28	X		0	21 June 2021	20 August 2021	16:00 to 00:00	X	X	X			
T8	F	16	X		0	29 June 2021	20 August 2021	7:00 to 15:30	X	X	X			

Note. ^1^ F: female; M: male.

## Data Availability

The data presented in this study are available on request from the corresponding author due to ethical reasons.
